# Supplementary material for: AddBiomechanics: Automating model scaling, inverse kinematics, and inverse dynamics from human motion data through sequential optimization
Source: bioRxiv. 2023 Jun 15:2023.06.15.545116. Preprint. [Version 1] doi: 10.1101/2023.06.15.545116 (PMC10312696; doi:10.1101/2023.06.15.545116)
Supplement: Supplementary file 1 [file NIHPP2023.06.15.545116v1-supplement-1.pdf]

bioRxiv preprint doi: https://doi.org/10.1101/2023.06.15.545116; this version posted June 15, 2023. The copyright holder for this preprint (which was not certified by peer review) is the author/funder, who has granted bioRxiv a license to display the preprint in perpetuity. It is made available under aCC-BY 4.0 International license.

Supporting information

S1 Appendix

Joint acceleration smoothing

Prior to finding inverse dynamics solutions with AddBiomechanics, we first perform a simple optimization to smooth the inverse kinematics solution we found by minimizing the jerk in the joint angle trajectories. This step is necessary to prevent large acceleration artifacts that appear as a result of small differences in joint angles between adjacent time steps from inverse kinematics. This optimization computes a new set of joint angles, $\hat{\boldsymbol{q}}_t$, and includes a regularization term controlled by the weighting parameter, σ, which prevents large deviations from the original inverse kinematics solution, $\boldsymbol{q}_t$.

$$\min_{\hat{\boldsymbol{q}}} \underbrace{||\frac{\partial^3}{\partial t^3}\hat{\boldsymbol{q}}_t||_2^2}_{\hat{\boldsymbol{q}}_t \text{ jerk}} + \underbrace{\sigma||\boldsymbol{q}_t - \hat{\boldsymbol{q}}_t||_2^2}_{\text{regularization}} \tag{23}$$

In our implementation, the joint jerks $\frac{\partial^3}{\partial t^3}\hat{\boldsymbol{q}}$ are computed using finite differences.

Constructing the $\tilde{\boldsymbol{b}}$ vector for angular dynamics fitting

The vector $\tilde{\boldsymbol{b}} \in \mathbb{R}^{6T}$ represents the current COM trajectory through space (first half, $3T$ entries), and the current root (e.g., pelvis) angular trajectory (second half, $3T$ entries), at the initial conditions $\boldsymbol{\zeta}$.

$$\tilde{\boldsymbol{b}} = \begin{bmatrix} \boldsymbol{z}_t \\ \boldsymbol{\theta}_t \end{bmatrix} \in \mathbb{R}^{6T} \tag{24}$$

To compute $\boldsymbol{z}_t$, note that we are holding the mass of the subject constant during this optimization step (m), so we can simply integrate the effects of known external forces (gravity, GRFs) on the known mass of the subject over time.

$$\ddot{\boldsymbol{z}}_t = \frac{\boldsymbol{f}_t}{m} - \boldsymbol{g} \tag{25}$$

$$\boldsymbol{z}_t = \boldsymbol{z}_1 + \sum_{i=1}^{t}(t-i)\Delta_t^2\ddot{\boldsymbol{z}}_i \tag{26}$$

To compute $\boldsymbol{\theta}_t$, we linearly integrate the "residual free angular acceleration" over time. We define the "residual free angular acceleration" with joint state $\boldsymbol{q}_t, \dot{\boldsymbol{q}}_t, \ddot{\boldsymbol{q}}_t$ to be the necessary root angular acceleration $\ddot{\boldsymbol{\theta}}_t$ such that no angular residual force is present. By convention $\ddot{\boldsymbol{\theta}}_t$ is always the first three entries of $\ddot{\boldsymbol{q}}_t$, which we write $\ddot{\boldsymbol{q}}_{t,[1:3]}$.

We can compute $\ddot{\boldsymbol{\theta}}_t$ given $\boldsymbol{q}_t, \dot{\boldsymbol{q}}_t, \ddot{\boldsymbol{q}}_t$ by first solving for joint torques $\boldsymbol{\tau}_t$ using inverse dynamics. Then, set the first three entries of $\boldsymbol{\tau}_t$ to 0, and solve forward dynamics using

bioRxiv preprint doi: https://doi.org/10.1101/2023.06.15.545116; this version posted June 15, 2023. The copyright holder for this preprint (which was not certified by peer review) is the author/funder, who has granted bioRxiv a license to display the preprint in perpetuity. It is made available under aCC-BY 4.0 International license.

$\boldsymbol{q}_t, \dot{\boldsymbol{q}}_t, \boldsymbol{\tau}_t$. The first three entries of the resulting $\ddot{\boldsymbol{q}}_t$ are the "residual free angular acceleration" $\ddot{\boldsymbol{\theta}}_t$.

Given the "residual free angular acceleration" $\ddot{\boldsymbol{\theta}}_t$, we can compute $\boldsymbol{\theta}_t$ by linearly integrating the "residual free angular acceleration" over time:

$$\boldsymbol{\theta}_t = \boldsymbol{\theta}_1 + \sum_{i=1}^{t} (t-i)\Delta_t^2 \ddot{\boldsymbol{\theta}}_i \tag{27}$$

Accounting for force plate registration errors

The location of experimental force plates are often registered incorrectly in the motion capture volume, which leads to solutions with center of mass trajectories offset slightly from the experimental marker data. To align our solution with the observed experimental data, we shift both the location of the force plates and the marker trajectories by the offset between the center of mass trajectory we find and the experimentally estimated center of mass trajectory, which typically is only a fraction of a centimeter.

It is also common to have ground reaction force data recorded from force plates that are very slightly (e.g., less than 0.5 degrees) off of perfectly vertical. This makes finding a physically-consistent solution challenging, because if we assume that the force plates are perfectly vertical in our optimization problem, the total ground reaction force vector will be very slightly off of perfectly vertical in the ground reference frame. This can lead to a substantial horizontal acceleration bias on very long trajectories, since the horizontal ground reaction forces will not be exactly anti-parallel to gravity.

Since the force plate rotation errors are typically very small, we can preserve the linearity of our system by using a first-order Taylor expansion to approximate the rotation of measured forces by very small angles. In our implementation, we append a rotational correction term $\boldsymbol{\alpha}_i \in \mathbb{R}^3$ to $\boldsymbol{\zeta}$ for each force plate i, where n is the number of force plates.

$$\boldsymbol{\zeta} = \begin{bmatrix} \boldsymbol{z}_1 \\ \dot{\boldsymbol{z}}_1 \\ \mu \\ \boldsymbol{\alpha}_1 \\ \vdots \\ \boldsymbol{\alpha}_n \end{bmatrix} \in \mathbb{R}^{7+3n} \tag{28}$$

For each $\boldsymbol{\alpha}_i$, we append to $\boldsymbol{A}$ a $3T \times 3$ columnar block:

$$\boldsymbol{A}_{\boldsymbol{\alpha}_i} = \begin{bmatrix} \boldsymbol{0} \\ \Delta_t^2[\boldsymbol{f}_1^i] \\ \Delta_t^2([\boldsymbol{f}_2^i] + 2[\boldsymbol{f}_1^i]) \\ \vdots \\ \Delta_t^2 \sum_{t=1}^{T} (T-t)[\boldsymbol{f}_t^i] \end{bmatrix} \tag{29}$$

where $\boldsymbol{f}_t^i$ is the ground reaction force vector associated with force plate i. The [.] operator makes a skew-symmetric matrix out of a vector in $\mathbb{R}^3$, so that $\boldsymbol{a} \times \boldsymbol{b} = [\boldsymbol{a}]\boldsymbol{b}$.

Finally, we regularize to angles $\boldsymbol{\alpha}_i$ to discourage large force plate rotations. With this extension to our linear fitting problem, we can recover force plate rotations to a very small fraction of a degree.

bioRxiv preprint doi: https://doi.org/10.1101/2023.06.15.545116; this version posted June 15, 2023. The copyright holder for this preprint (which was not certified by peer review) is the author/funder, who has granted bioRxiv a license to display the preprint in perpetuity. It is made available under aCC-BY 4.0 International license.

Quantifying the impact of using the bilevel constraint in kinematics fitting

We ran an ablation study to quantify the impact of the formulating our kinematics fitting problem as a bilevel optimization problem. Here, we compare two conditions: optimizing a bilevel objective and optimizing a simple monolevel objective. The objective for the bilevel optimization problem (stated previously in Eq (2)) is as follows:

$$\max_{\boldsymbol{s},\boldsymbol{p}} \left(\max_{\boldsymbol{q}_t} \; P_{\bar{x}}(\bar{\boldsymbol{x}}_t|\boldsymbol{q}_t, \boldsymbol{s}, \boldsymbol{p}) \cdot P_s(\boldsymbol{s}) \cdot P_p(\boldsymbol{p}|\bar{\boldsymbol{p}}) \right) \tag{30}$$

To convert the bilevel objective to a monolevel objective, we move joint angle optimization, $\max_{\boldsymbol{q}_t}$, to the outer objective along with the body scale and marker registration optimization:

$$\max_{\boldsymbol{s},\boldsymbol{p},\boldsymbol{q}_t} \left(P_{\bar{x}}(\bar{\boldsymbol{x}}_t|\boldsymbol{q}_t, \boldsymbol{s}, \boldsymbol{p}) \cdot P_s(\boldsymbol{s}) \cdot P_p(\boldsymbol{p}|\bar{\boldsymbol{p}}) \right) \tag{31}$$

We find that using a bilevel approach leads to much faster convergence, and we can get lower marker RMSE at the cost of slightly lower anthropometric prior probability. The bilevel optimizer takes slightly more wall-clock time per iteration, but because it is able to reach high quality marker RMSE in many fewer iterations, it is able to save wall-clock time overall. We ran both optimization functions for several different fixed numbers of iterations on a single walking trial on a commodity server; these results are summarized in Table A1.

Table A1. Monolevel versus bilevel optimization.

	**Monolevel**			**Bilevel**		
Iterations	Time (s)	RMSE (m)	Anthro†	Time (s)	RMSE (m)	Anthro†
100	6.817	0.0166	26.6	10.6	0.0150	25.9
300	21.693	0.0203	26.8	31.9	0.0150	26.6
500	35.7	0.0153	26.9	52.6	0.0146	26.9
1000	70.3	0.0157	27.5	104	0.0144	27.4
2000	178	0.0157	27.5	233	0.014523	27.5

† This is the probability density function value for a multivariate gaussian derived from the ANSUR II dataset [55] of human anthropometrics.
